# Predicting outcomes in locally advanced cervical cancer treated with radiotherapy: a systematic review of nomograms

**DOI:** 10.3389/fonc.2026.1940047

**Published:** 2026-10-22

**Authors:** Jaekyung Bae, Uisuk Kim, Sokbom Kang

**Affiliations:** 1 Center for Gynecologic Cancer, National Cancer Center, Goyang, Republic of Korea; 2 Department of Cancer Control and Policy, Graduate School of Cancer Science and Policy, National Cancer Center, Goyang, Republic of Korea

**Keywords:** cervical cancer, nomogram, prognosis, radiotherapy, systematic review

## Abstract

**Objective:**

To systematically evaluate published nomograms developed for patients with locally advanced cervical cancer (LACC) treated with radiotherapy, focusing on prognostic performance, core predictive variables, and implications for future model development.

**Methods:**

A systematic literature search of PubMed, Embase, Scopus, Web of Science, and the Cochrane Library identified studies published between 2010 and 2025 that developed new nomograms for LACC cohorts treated with radiotherapy or chemoradiotherapy. Data extraction included study population, predicted outcomes (overall survival [OS], progression-free survival [PFS], or disease-free survival [DFS]), model performance (C-index), validation methods, calibration, and predictor variables. Predictor patterns were summarized primarily by frequency of inclusion across nomograms, with β-coefficients used only for exploratory within-model descriptions.

**Results:**

Nine studies met the inclusion criteria; eight developed nomograms for OS and three evaluated disease progression outcomes (PFS or DFS), with two studies evaluating both outcome categories. Reported C-indices ranged from 0.68 to 0.835, indicating moderate to good discrimination. Lymph node status, FIGO stage, and tumor size were among the most frequently incorporated predictors, while histology and SCC-Ag were also recurrently included. Validation in independent cohorts remained limited. Graphical calibration was commonly reported, whereas quantitative calibration measures and decision-curve analyses were inconsistently reported.

**Conclusions:**

Nomograms for LACC treated with radiotherapy generally demonstrate moderate-to-good discriminative performance and frequently incorporate lymph node status, stage, and tumor size. However, methodological heterogeneity, incomplete validation, and evolving treatment paradigms limit their immediate generalizability. Future work should focus on robust external validation, recalibration, and evaluation in contemporary treatment cohorts before clinical implementation.

## 1 Introduction

Cervical cancer remains a major global public health concern. In 2022, an estimated 662,044 new cases (age-standardized incidence rate: 14.12 per 100,000) and 348,709 deaths (age-standardized mortality rate: 7.08 per 100,000) were reported, making it the fourth leading cause of cancer incidence and mortality among women worldwide (1). For women with locally advanced cervical cancer (LACC), concurrent chemoradiotherapy combined with brachytherapy remains the standard of care. However, clinical outcomes vary considerably depending on disease stage, tumor biology, treatment modality, and patient-related factors. This heterogeneity highlights the need for reliable predictive tools to support individualized treatment decisions.

In this context, nomograms have emerged as practical prognostic tools that integrate multiple clinical and pathological factors to provide individualized risk estimates. Several studies have developed nomograms predicting overall survival (OS) and disease progression outcomes, including progression-free survival (PFS) and disease-free survival (DFS) in LACC patients receiving radiotherapy or chemoradiotherapy (2–10).

The objective of this systematic review is to provide a comprehensive evaluation of currently available nomograms for LACC treated with radiotherapy. In particular, we focus on nomograms predicting oncological outcomes such as OS, PFS, and DFS. By synthesizing existing evidence, this review aims to critically evaluate their methodological characteristics and identify requirements for future validation and clinical implementation.

## 2 Materials and methods

### 2.1 Evidence acquisition

We conducted a systematic literature search to identify studies reporting the development of nomograms for patients with LACC undergoing radiotherapy, published between January 2010 and April 2025. The primary database was PubMed, with complementary searches in Embase, Scopus, Web of Science, and the Cochrane Library to ensure comprehensive coverage. Searches were last updated on April 30, 2025. The search strategy combined four concepts: nomograms, cervical cancer, locally advanced disease, and radiotherapy-based treatment. The following search strategy was used across all five databases:

(“Nomogram” OR “Nomograms”) AND (“Cervical cancer” OR “Cervix cancer” OR “Cervix carcinoma”) AND (“Locally advanced”) AND (“Radiotherapy” OR “Radiation therapy” OR “Chemoradiotherapy” OR “Brachytherapy”). The search was limited to English-language articles.

### 2.2 Eligibility criteria

We included human studies that developed new prognostic nomograms for patients with LACC receiving radiotherapy as a major component of treatment, including definitive RT/CCRT and mixed cohorts containing patients treated with surgery followed by adjuvant radiotherapy, and that reported OS, PFS or DFS with measures of model performance. PFS and DFS were retained as distinct endpoints and were not assumed to be interchangeable; however, because only three studies evaluated disease progression outcomes, they were grouped as PFS/DFS for descriptive synthesis. We excluded case reports, editorials, letters, and commentaries. Abstract-only publications, such as conference abstracts without full text, and studies that only validated previously published nomograms without reporting new model development were also excluded. For the purposes of this review, we did not impose a single FIGO stage-based definition of LACC because the terminology, stage ranges, and FIGO staging editions varied across the original studies. Eligible studies were those described by the original investigators as involving LACC or populations broadly corresponding to locally advanced disease treated with radiotherapy. The stage range and FIGO staging framework used in each study were extracted and reported individually. We therefore did not retrospectively reclassify all study populations according to the FIGO 2018 staging system.

### 2.3 Study selection

Titles and abstracts were independently screened by two reviewers and classified as irrelevant, relevant, or uncertain. Inter-reviewer agreement for the initial screening was assessed using Cohen’s kappa coefficient. Across all 234 records, exact agreement between the two reviewers was 79.9%, with a Cohen’s κ of 0.65. Discrepant or uncertain assessments were subsequently reviewed and resolved by consensus. The study selection process is summarized in a PRISMA flow chart, illustrating the number of records identified, screened, excluded, and included (**Figure 1**).

**Figure 1:**
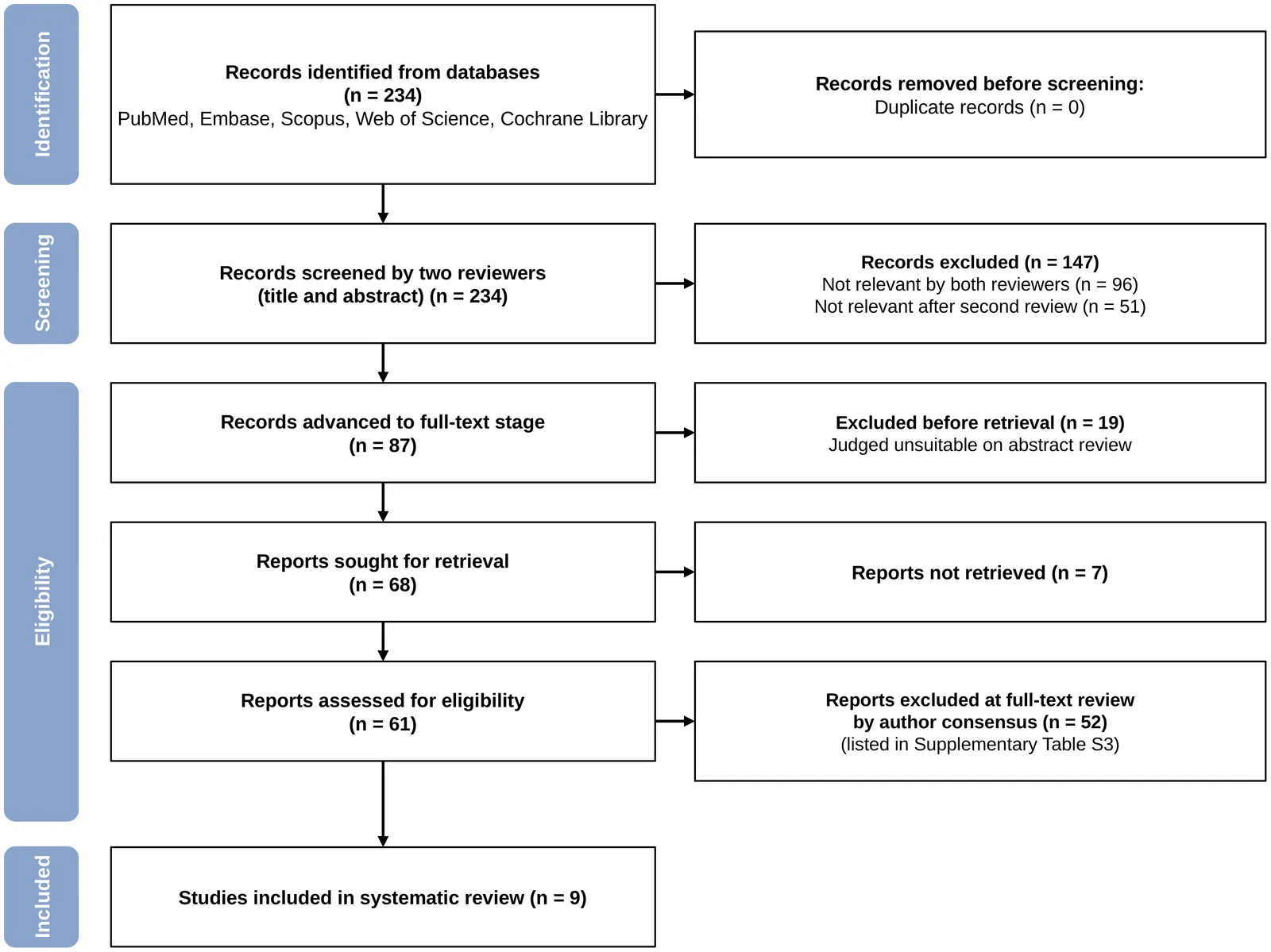
PRISMA 2020 flow diagram of the study selection process.

### 2.4 Data extraction and quality assessment

From each eligible study, we extracted patient characteristics (stage, histology, and treatment setting), predicted outcome (OS, PFS, or DFS), total and development-sample size, validation-sample size and source, number of outcome events when explicitly reported, median follow-up, missing-data handling, number of candidate and final predictor parameters when available, model-development approach, discrimination (C-index and/or AUC), validation method, graphical calibration, quantitative calibration measures (calibration intercept, calibration slope, observed-to-expected ratio, and calibration error), decision-curve analysis, and final predictor variables. Information not explicitly reported in the original publication was recorded as not reported and was not retrospectively estimated. C-index values were interpreted as follows: 0.5 indicated no discriminative ability, values between 0.7 and 0.8 reflected acceptable discrimination, and values below 0.7 were considered relatively low. The methodological rigor and predictive performance of each nomogram were evaluated according to their primary predicted outcomes (OS and PFS/DFS).

To characterize predictor patterns across studies, we used two complementary descriptive approaches. First, we quantified the frequency with which each predictor was included across the identified nomograms; this frequency was used as the primary cross-study descriptive measure. Second, for models reporting regression coefficients, we calculated the absolute β coefficient as a proportion of the sum of absolute β coefficients within the same model. Because β coefficients depend on predictor scaling, coding, reference categories, categorization, and model specification, these proportions were considered exploratory within-model descriptive measures only. They were not interpreted as standardized measures of predictor importance or compared quantitatively across different models.

Risk of bias (RoB) and applicability were independently assessed by two reviewers using the Prediction model Risk Of Bias ASsessment Tool (PROBAST) (11).

All 20 signaling questions across the four RoB domains (participants, predictors, outcome, and analysis) were reassessed for each included study. Signaling questions were rated as yes, probably yes, probably no, no, or no information according to the information reported in the original publications. Domain-level and overall RoB judgments were then derived from the item-level assessments. When reporting was insufficient to support a confident judgment, the relevant item was classified as no information rather than assumed to be low risk. Detailed item-level assessments, domain-level judgments, and supporting model-development characteristics are provided in **Supplementary Tables S1**–**S3**.

Given heterogeneity in predictors, outcome time points, and reporting of performance metrics, we conducted a narrative synthesis without meta-analysis. The review protocol was not registered.

## 3 Results

### 3.1 Characteristics of included studies

Titles and abstracts of 234 records were independently screened by two reviewers, of which 147 were excluded and 87 advanced to the full-text retrieval stage. Nineteen reports were excluded before retrieval following additional assessment, and seven reports could not be retrieved, leaving 61 reports for full-text eligibility assessment. Of these, 52 were excluded by author consensus and are listed in **Supplementary Table S4**. The remaining nine studies met all eligibility criteria and were included in the final systematic review. The study selection process is summarized in **Figure 1**.

The nine included studies enrolled cervical cancer populations broadly representing locally advanced disease, although the stage ranges and FIGO staging frameworks varied across studies. Among the nine included studies, eight developed nomograms for OS and three developed nomograms for disease progression outcomes, including PFS or DFS; two studies evaluated both outcome categories. Meng et al. specifically reported DFS rather than PFS. For comparative analysis, studies were therefore categorized into OS and PFS/DFS groups (**Tables 1**, **2**).

**Table 1:** Nomograms predicting overall survival.

Author	Year	FIGO stage	Histology	Treatment 1. Surgery + adjuvant RT 2. Surgery (including LND) + adjuvant RT 3. RT only (EBRT and BT) (chemotherapy or not)	Outcome/endpoint	Prediction time point	Sample size	AUC	C-index	Validation type/cohort	Calibration	Variables	Model type
Lin Y	2024	IB2-IVA	Squamous cell carcinoma, adenocarcinoma, others	CCRT	Overall survival	1, 2, 3 year	582 (training 228; internal validation 98; external validation 256)	0.809 (1-year) 0.808 (2-year) 0.862 (3-year)	0.783	internal, external	present	Age, Parametrial invasion, Radiomic score	Cox
Hu J	2024	IB-IVA	Squamous cell carcinoma, non- Squamous cell carcinoma	Definitive RT (n=147); Surgery followed by adjuvant RT (n=141)	Overall survival	3, 5 year	288 (development); 74 (external validation)	0.849 (3-year) 0.840 (5-year)	0.815	external	present	Age, Anemia, Acute radiation enteritis, Comorbidity, Differentiation, LN status, Neutrophil-to-lymphocyte ratio, Stage	Cox
Ma H	2025	IB1-IVA	Squamous cell carcinoma, Non- Squamous cell carcinoma	Radical RT ± concurrent chemotherapy	Overall survival	3 year	159	0.695 (Nomogram 1) 0.822 (Nomogram 2)	0.709 (Nomogram 1) 0.803 (Nomogram 2)	internal	present	Nomogram 1: Stage, Pre-treatment albumin Nomogram 2: Chemotherapy, Minimum neutrophil during treatment, Lymphopenia grade, Pre-treatment albumin, Rate of tumor shrinkage before brachytherapy, Stage,Treatment times	Cox
Meng Q	2022	IB-IVA	Squamous cell carcinoma, Non- Squamous cell carcinoma	RT	Overall survival	3, 5 year	1,192 (development 795; validation 397)	0.81 (3-year) 0.81 (5-year)	0.78	internal	present	Age, Histology, LN status, Stage, SCC-Ag	Cox
Zhang K	2023	IB2-IVA	Squamous cell carcinoma, adenocarcinoma, others	RT	Overall survival	5 year	238 (training 165; test 73)	Test cohort 0.729 (clinical) 0.703 (pathomic) 0.750 (clinical-pathomic)	Training cohort 0.68 (clinical) 0.74 (pathomic) 0.83 (clinical-pathomic)	internal	present	Clinical: Tumor size, LN status Pathomic: Deep feature Clinical-pathomic: Deep feature, LN status, Tumor size	Cox
Zang L	2021	II-III	Squamous cell carcinoma	RT	Overall survival	3, 5 year	469	0.72 (3 year) 0.73 (5 year)	0.71	Internal	present	LN metastasis, Lower 1/3 vaginal involvement, Tumor length, Tumor width	Cox
Zang L	2023	II-IIIC2r	Squamous cell carcinoma	RT	Overall survival	3, 5, 7 year	396 (training 277; validation 119)	0.67 (3-year) 0.68 (5-year) 0.71 (7-year)	0.74	Internal	present	Chemotherapy, Differentiation, SCC-Ag, Stage, Tumor size	Cox
Yang X	2020	IB-IVA	Squamous cell carcinoma	RT	Overall survival	5 year	613	0.692	0.713	internal	present	BMI, PALN metastasis, Pelvic wall involvement, SCC-Ag, Tumor size	Cox

FIGO, International Federation of Gynecology and Obstetrics; RT, Radiation therapy; LND, Lymph node dissection; EBRT, External beam radiation therapy; BT, Brachytherapy; AUC, Area under curve; CCRT, Concurrent chemoradiation therapy; LN, Lymph node; BMI, Body mass index; PALN, Paraaortic lymph node.

**Table 2:** Nomograms predicting progression-free survival or disease-free survival.

Author	Year	FIGO stage	Histology	Treatment 1. Surgery + adjuvant RT 2. Surgery (including LND) + adjuvant RT 3. RT only (EBRT and BT) (chemotherapy or not)	Outcome/endpoint	Prediction time point	Sample size	AUC	C-index	Validation type/cohort	Calibration	Variables	Model type	Risk of Bias	Applicability concern
Meng Q	2022	IB-IVA	Squamous cell carcinoma, Non- Squamous cell carcinoma	RT	Disease-free survival*	3,5 year	1,192 (development 795; validation 397)	0.77 (3-year) 0.73 (5-year)	0.74	internal	present	Histology, PLN number, SCC-Ag, Stage	Cox	low	low
Luo WX	2024	IIIC1r	Squamous cell carcinoma	- CCRT (n=121) - Radical hysterectomy followed by radiotherapy and chemotherapy (n=23)	Progression-free survival	1,3 year	144 (training 100; independent validation 44)	training 1-y = 0.891 training 3-y = 0.921 validation 1-y = 0.902 validation 3-y = 0.885	training = 0.835; independent validation = 0.789	external (independent validation cohort, n=44)	present	Stage, Tumor size, Short diameter of the maximum metastatic LN, roundness index of the maximum metastatic LN	Cox	low	low
Yang X	2020	IB-IVA	Squamous cell carcinoma	RT	Progression-free survival	5 year	613	0.687	0.686	internal	present	BMI, Hb, PaLN metastasis, PLN metastasis, Pelvic wall involvement, Tumor size	Cox	low	low

FIGO, International Federation of Gynecology and Obstetrics; RT, Radiation therapy; LND, Lymph node dissection; EBRT, External beam radiation therapy; BT, Brachytherapy; AUC, Area under curve; PLN, Pelvic lymph node; CCRT, Concurrent chemoradiation therapy; PFS, Progression-free survival; LN, Lymph node; BMI, Body mass index; Hb, Hemoglobin; PaLN, Paraaortic lymph node.

^*^ Meng et al. reported disease-free survival (DFS) as the study endpoint; this outcome is presented alongside PFS models in this review for descriptive comparison.

Patient cohorts varied in stage distribution, histologic subtype, and treatment modality. While squamous cell carcinoma was the predominant histology, adenocarcinoma and other non-squamous types were also represented. Most studies evaluated patients treated with definitive RT or CCRT, whereas Hu et al. and Luo et al. included mixed-treatment populations containing patients who underwent surgery followed by adjuvant radiotherapy. Model-development approaches also varied: most studies used multivariable Cox regression, whereas radiomic or pathomic studies additionally incorporated high-dimensional feature extraction and penalized feature-selection procedures. Validation strategies ranged from bootstrap resampling and random split-sample validation to evaluation in cohorts distinct from the development sample. Lin et al. used an independent multicenter external cohort, Luo et al. used an independent cohort from a second institution, and Hu et al. evaluated the model in a temporally separate cohort from the same hospital. Graphical calibration was frequently presented, but calibration intercepts, calibration slopes, observed-to-expected ratios, and calibration errors were generally not reported. Sample sizes ranged widely, from 144 to 1192 patients. This treatment heterogeneity may contribute to differences in baseline risk and outcome distributions and should be considered when interpreting and comparing model performance across studies. Additional model-development characteristics are summarized in **Supplementary Table S3**.

### 3.2 Risk of bias and applicability assessment

The detailed PROBAST assessment is presented in **Supplementary Tables S1** and **S2**, with supporting model-development characteristics in **Supplementary Table S3**. Methodological concerns were concentrated primarily in the Analysis domain. Recurrent issues included incomplete reporting of outcome-event adequacy and missing-data handling, data-driven categorization of continuous predictors, predictor selection based on univariable screening, and limited adjustment for model optimism in several studies. In addition, blinding of outcome determination to predictor information was generally not reported, resulting in uncertainty in the Outcome domain despite the use of standard time-to-event definitions. Applicability concerns were greatest for models requiring specialized radiomic or pathomic feature extraction and were less pronounced for models based predominantly on routinely available clinical variables. These findings indicate that the reported discrimination of the included nomograms should be interpreted alongside methodological limitations in model development and validation.

### 3.3 Nomograms predicting OS

Eight studies developed nomograms for OS prediction (**Table 1**). Six studies included patients treated with definitive radiotherapy (4–9). Lin et al. included only patients who received CCRT, while Hu et al. included both definitive CCRT patients and those who underwent surgery followed by adjuvant radiotherapy (2, 10). Sample sizes ranged from 159 to 795 patients.

The reported C-indices ranged from 0.68 to 0.83, indicating moderate to good discriminative performance. Hu et al. reported a C-index of 0.815 and evaluated the model in a temporally separate cohort from the same hospital, whereas Lin et al. evaluated their model in an independent multicenter external cohort. Zhang et al. reported a C-index of 0.68 for the clinical model, which increased to 0.83 when clinical and pathomic information were combined (2, 6). Among the eight OS nomogram studies, Lin et al. performed independent multicenter external validation, whereas Hu et al. performed temporal validation using a later-period cohort from the same hospital. Prognostic variables commonly used in OS nomograms included tumor stage, histology, lymph node status, parametrial invasion, and serological markers such as SCC-Ag. Some models also incorporated patient-related factors such as age, comorbidity, anemia, serum albumin level, and radiomics scores, reflecting attempts to improve individualized prediction. Reported area under curve (AUC) values further supported the discriminative ability, with four studies achieving AUCs > 0.80 and three achieving AUCs > 0.70 for 3-, 5-, or 7-year OS prediction.

### 3.4 Nomograms predicting PFS or DFS

Three studies developed nomograms predicting PFS or DFS (**Table 2**). Among these, Yang et al. and Luo et al. evaluated PFS, whereas Meng et al. reported DFS as the study endpoint. Two studies included patients treated with definitive radiotherapy, whereas Luo et al. included both patients who received definitive CCRT and those who underwent surgery followed by adjuvant radiotherapy (3, 4, 9).

The reported C-indices ranged from 0.686 to 0.835. Luo et al. achieved the highest performance, with a C-index of 0.835 and AUCs of 0.891 at 1-year and 0.921 at 3-years (3). In comparison, Yang et al.’s model demonstrated relatively lower predictive accuracy, with a C-index of 0.686 (9). Among the three nomogram studies, Luo et al. evaluated their model in an independent validation cohort from a second institution. Variables commonly included in PFS/DFS nomograms were tumor stage, tumor size, SCC-Ag levels, and lymph node parameters such as pelvic lymph node number, para-aortic involvement, and metastatic lymph node short-axis diameter.

### 3.5 Predictor frequency and within-model coefficient patterns

Predictor variables were summarized primarily according to their frequency of inclusion across nomograms (**Figure 2**), with β-derived proportions used only as exploratory within-model descriptions (**Tables 3**, **4**). For OS, lymph node status, tumor stage, and tumor size were among the most frequently incorporated predictors (**Figure 2A**). In Hu et al., pelvic lymph node metastasis was associated with OS (HR 3.595, 95% CI 2.109–6.128; β=1.28), representing 17.8% of the summed absolute β coefficients within that model (2). In Zhang et al.’s model, lymph node metastasis alone accounted for 43.1% of the total β within the model (6). Tumor stage also showed a consistent effect: advanced stage (III–IV) carried an HR of 1.947 in Hu et al.’s study and 1.502 in Ma et al.’s study, accounting for approximately 9% of the total β within the respective models (**Table 3**).

**Figure 2:**
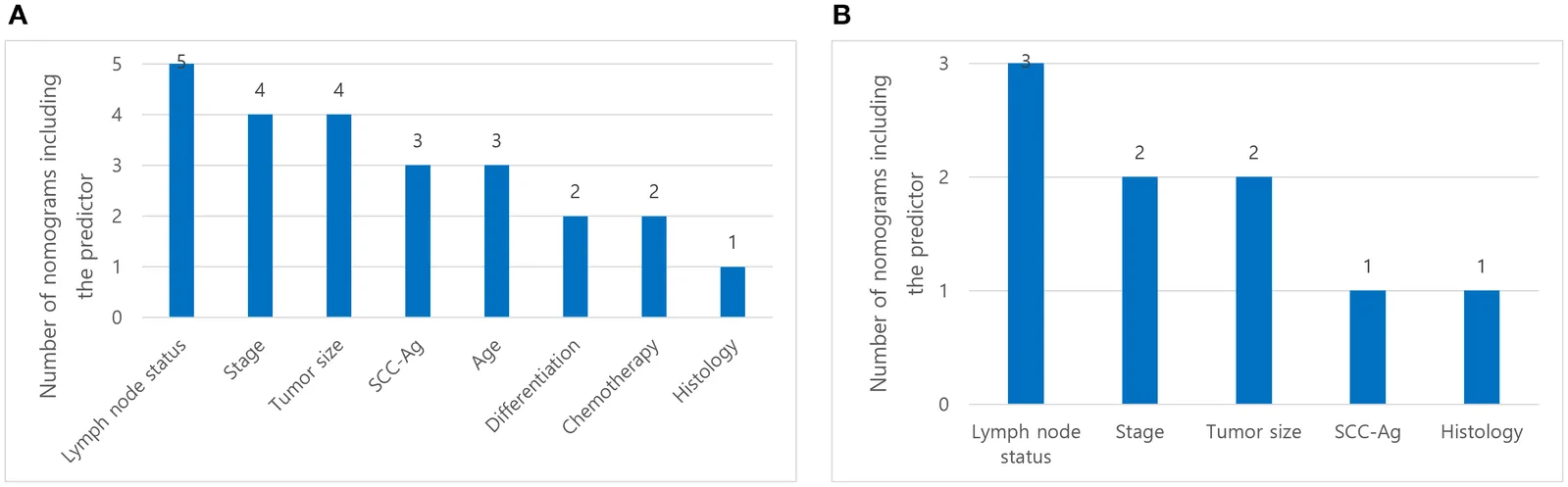
Frequency of predictor inclusion across prognostic nomograms. Number of published nomograms incorporating each predictor for **(A)** overall survival and **(B)** progression-free survival or disease-free survival. Predictor frequency represents the number of models in which the variable was included and should not be interpreted as a standardized measure of predictor importance or effect size.

**Table 3:** Reported effect estimates and within-model coefficient patterns for overall survival.

Prognostic factor	Author	C-index	Variables	HR (95% CI)	β=ln(HR)	Within-model share of absolute β (%)
LN status	Hu J	0.815	PLN meta	3.595 (2.109-6.128)	1.28	17.75%
	Meng Q	0.78	PLN number 0-1	Reference	–	–
			PLN number >=2	1.57 (1.15-2.15)	0.451	4.35%
	Zhang K	0.83	LN meta	2 (0.67-5.8)	0.693	43.11%
	Zang L	0.71	LN meta	1.96 (1.27-3.02)	0.673	30.22%
	Yang X	0.713	PALN meta	2.46 (1.408-4.297)	0.9	23.95%
FIGO Stage	Hu J	0.815	III-IV	1.947 (1.075-3.529)	0.666	9.24%
	Ma H	0.803	Stage (continuous)	1.502 (0.732-3.081)	0.407	8.90%
	Meng Q	0.78	IB	Reference	–	–
			IIA	1.24 (0.33-4.62)	0.215	2.08%
			IIB	1.14 (0.43-3.01)	0.131	1.26%
			IIIA	4.09 (1.07-15.59)	1.409	13.59%
			IIIB	1.87 (0.53-6.61)	0.626	6.04%
			IIIC1	2.26 (0.78-6.56)	0.815	7.87%
			IIIC2	5.77 (1.89-17.67)	1.753	16.91%
			IVA	11.06 (2.51-48.83)	2.403	23.18%
	Zang L	0.74	II	Reference	–	–
			IIIA, IIIB	1.42 (0.55-3.70)	0.351	5.74%
			IIIC1r	2.45 (1.07-5.60)	0.896	14.66%
			IIIC2r	4.26 (1.49-12.17)	1.449	23.71%
Tumor size	Zhang K	0.83	Tumor volume (continuous)	1.2 (0.93-1.7)	0.182	11.34%
	Zang L	0.71	Tumor length > 5.1cm	1.77 (1.06-2.94)	0.571	25.65%
			Tumor width > 4.13cm	1.68 (1.03-2.76)	0.519	23.30%
	Zang L	0.74	Tumor volume > 28.8cm3	2.84 (1.40-5.74)	1.044	17.07%
	Yang X	0.713	Tumor size <= 4cm	Reference	–	–
			Tumor size 4.1~5.9 cm	1.785 (1.088-2.929)	0.579	15.42%
			Tumor size >= 6cm	1.934 (1.072-3.491)	0.66	17.55%

HR, Hazard ratio; CI, Confidence interval; LN, Lymph node; PLN, Pelvic lymph node; PALN, Paraaortic lymph node; FIGO, International Federation of Gynecology and Obstetrics.

β-derived percentages represent the relative magnitude of individual coefficients within each original model and should not be interpreted as standardized measures of predictor importance across different models because β coefficients depend on variable scaling, coding, and model specification.

**Table 4:** Reported effect estimates and within-model coefficient patterns for progression-free survival or disease-free survival.

Prognostic factor	Author	C-index	Variables	HR (95% CI)	β=ln(HR)	Within-model share of absolute β (%)
LN status	Meng Q^*^	0.74	PLN number 0-1	Reference	–	–
			PLN number >= 2	1.56 (1.21-2.02)	0.445	5.18%
	Luo WX	0.835	LN short diameter <= 1cm	Reference	–	–
			LN short diameter > 1cm	4.004 (1.321-12.132)	1.387	20.53%
			Roundness index of the maximum metastatic LN >= 2	Reference	–	–
			Roundness index of the maximum metastatic LN <2	6.369 (1.424-28.492)	1.851	27.40%
	Yang X	0.686	PLN meta	1.49 (1.050-2.114)	0.399	14.91%
			PALN meta	3.266 (2.047-5.208)	1.184	44.27%
FIGO Stage	Meng Q^*^	0.74	IB	Reference	–	–
			IIA	1.31 (0.49-3.53)	0.27	3.15%
			IIB	1.36 (0.67-2.78)	0.307	3.58%
			IIIA	2.36 (0.78-7.13)	0.859	10.01%
			IIIB	2.21 (0.90-5.43)	0.793	9.24%
			IIIC1	1.69 (0.75-3.82)	0.525	6.12%
			IIIC2	4.81 (2.02-11.47)	1.571	18.31%
			IVA	8.47 (2.52-28.52)	2.137	24.90%
	Luo WX	0.835	IB-IIA^†^	Reference	–	–
			IIB-IIIB^†^	4.806 (1.509-15.304)	1.57	23.23%
Tumor size	Luo WX	0.835	Tumor size <=4cm	Reference	–	–
			tumor size >4cm	7.024 (1.421-34.709)	1.949	28.85%
	Yang X	0.686	Tumor size <=4cm	Reference	–	–
			Tumor size 4.1~5.9 cm	1.401 (0.967-0.996) ^‡^	0.337	12.61%
			Tumor size ≥ 6 cm	1.063 (0.660-1.713)	0.061	2.28%

HR, Hazard ratio; LN, lymph node; PLN, pelvic lymph node; PALN, paraaortic lymph node; FIGO, International Federation of Gynecology and Obstetrics.

β-derived percentages represent the relative magnitude of individual coefficients within each original model and should not be interpreted as standardized measures of predictor importance across different models because β coefficients depend on variable scaling, coding, and model specification.

^*^ Meng et al. reported disease-free survival (DFS) as the study endpoint; this outcome is presented alongside PFS models in this review for descriptive comparison.

^†^ In Luo et al., the stage variable included in the prognostic model was based on the 2009 FIGO staging system (IB–IIA vs. IIB–IIIB), whereas the study population comprised patients with FIGO 2018 stage IIIC1r disease.

^‡^ The HR and 95% CI for tumor size 4.1–5.9 cm in Yang et al. are reproduced as reported in the original publication; the reported HR falls outside the stated 95% CI.

For PFS/DFS, lymph node status, tumor stage, and tumor size were the most frequently included predictors (**Figure 2B**). Luo et al. reported that metastatic nodes >1 cm in short-axis diameter were associated with worse PFS (HR 4.004, 95% CI 1.321-12.132, β=1.387), accounting for 20.5% of the total β within the model (3) (**Table 4**). In the same study, the roundness index of the maximum metastatic node showed a relatively large effect estimate (HR 6.369, 95% CI 1.424-28.492, β=1.851), and represented 27.4% of the summed absolute β coefficients within the model. Meng et al. reported that ≥2 positive pelvic lymph nodes were associated with worse DFS (HR 1.56, 95% CI 1.21–2.02; β=0.445), representing 5.2% of the summed absolute β coefficients within that model (4). Overall, lymph node status, FIGO stage, and tumor size were among the most frequently incorporated predictors across OS and PFS/DFS nomograms. The β-derived percentages should be interpreted only as descriptive measures of coefficient magnitude within individual models and not as standardized or directly comparable measures of predictor importance across studies.

## 4 Discussion

### 4.1 Summary of the main results

In this systematic review of nine studies developing prognostic nomograms for patients with LACC treated with radiotherapy, overall discriminative performance was moderate to good, with reported C-indices ranging from 0.68 to 0.835. Lymph node status, stage, and tumor size were among the most frequently incorporated predictors, while histology and SCC-Ag were also recurrently included. Exploratory examination of β-coefficients showed relatively large effect estimates for several lymph node-related variables within individual models. However, because β-derived proportions depend on predictor scale, coding, and model specification, they should be interpreted only as within-model descriptive measures and not as standardized measures of predictor importance across studies. Overall, considerable heterogeneity in patient populations, treatment settings, staging systems, predictor definitions, and validation strategies limits direct comparison across existing nomograms.

### 4.2 Evolving treatment landscape in LACC and implications for modeling

For patients with LACC, CCRT with brachytherapy remains the standard of care. However, recent pivotal trials have reshaped the therapeutic landscape. The phase III INTERLACE trial enrolled patients with LACC (FIGO 2008 stage IB1 with nodal involvement and stage IB2–IVA) and demonstrated that short-course induction chemotherapy prior to CCRT significantly improved 5-year PFS and OS compared with CCRT alone, underscoring its potential impact on baseline risk and treatment pathways in contemporary practice (12).

Similarly, the KEYNOTE-A18 trial (ENGOT-cx11/GOG-3047) evaluated high-risk LACC patients (FIGO 2014 stage III–IVA or stage IB2–IIB with nodal metastasis) and showed that adding pembrolizumab to CCRT, followed by maintenance immunotherapy, significantly improved 3-year OS. These results support immuno-chemoradiotherapy as a new standard of care for appropriately selected patients (13).

In parallel, advances in radiation technology have changed local therapy. MRI-guided adaptive brachytherapy (IGABT), as evaluated in the multicenter prospective EMBRACE-I study, provided improved target coverage and sparing of normal tissues. Long-term outcomes demonstrated excellent local control (~92% at 5 years) with acceptable late toxicity, establishing IGABT as a benchmark for brachytherapy in LACC (14).

Collectively, these developments highlight the growing heterogeneity of LACC treatment, with systemic therapy intensification and technological advances influencing survival outcomes and event rates. Against this backdrop, robust and generalizable risk stratification tools are essential. Our systematic review provides an evidence-based synthesis of core predictors and performance benchmarks to inform future model development, recalibration, and clinical decision-making in the era of evolving treatment paradigms.

An additional source of heterogeneity was variation in treatment setting and staging systems across the included studies. Although most nomograms were developed in patients receiving definitive RT or CCRT, two studies included mixed populations containing patients treated with surgery followed by adjuvant RT. These populations may differ systematically from definitive RT cohorts because surgical selection, pathological staging, lymph-node assessment, and postoperative treatment indications can influence baseline prognosis and the apparent prognostic contribution of individual predictors. Furthermore, the original studies spanned different FIGO staging eras and treatment periods. Consequently, nominally similar stage categories may not represent identical disease-risk groups across studies, particularly following the incorporation of nodal status into FIGO 2018 stage IIIC. These differences limit direct comparisons of model performance and predictor effects across nomograms.

### 4.3 Core prognostic variables for LACC nomograms

Our synthesis identified five variables that were recurrently incorporated into published nomograms for patients with LACC treated with radiotherapy (**Figure 2**; **Tables 3**, **4**): (1) lymph node characteristics, including site, number, or size; (2) FIGO stage; (3) tumor size; (4) histology; and (5) SCC-Ag. Among these, lymph node characteristics, FIGO stage, and tumor size were the most frequently represented across OS and PFS/DFS models. Histology and SCC-Ag were also incorporated in several models, although their inclusion varied across cohorts. Because effect estimates depend on predictor coding, scaling, and model specification, the relative prognostic importance of these variables cannot be directly ranked across studies. These recurrent variables may nevertheless provide a useful set of candidate predictors for future model development and validation.

### 4.4 Methodological considerations and analytical planning

Building on the findings of this review, recurrent variables such as lymph node characteristics, FIGO stage, tumor size, histology, and SCC-Ag may be considered as candidate predictors in future model development, together with additional variables supported by clinical relevance and contemporary evidence. Careful attention to sample size and outcome-event planning is essential to reduce overfitting and improve the precision of absolute risk estimates. Rather than relying solely on a fixed events-per-variable rule, future studies should justify sample size using prediction-model-specific methods that account for the anticipated outcome incidence, the number of candidate predictor parameters, expected model fit, and a target level of shrinkage or optimism.

Future studies should therefore justify sample size using prediction-model-specific methods, such as those proposed by Riley et al. (15, 16), rather than relying solely on a fixed events-per-variable threshold.

The broader applicability of nomogram-based prognostic modeling is also illustrated in other oncologic settings. For example, La Salvia et al. recently developed the Rachel score for lung neuroendocrine tumors, integrating multiple prognostic variables into a nomogram that stratified patients into distinct prognostic groups (17). Although developed in a different tumor type, this study illustrates the potential of nomograms to translate multivariable prognostic information into individualized risk estimates. Importantly, however, such tools remain disease- and population-specific, and their clinical relevance depends on appropriate validation, calibration, and demonstration of utility in the intended target population.

### 4.5 Model performance, validation, and clinical utility

Discrimination should be distinguished from calibration and clinical utility when interpreting prognostic nomograms. Although most included studies presented calibration plots, quantitative calibration measures such as the calibration intercept, calibration slope, observed-to-expected ratio, and calibration error were generally not reported. Decision-curve analysis was reported in only a subset of studies, and the nature of validation varied substantially. Lin et al. and Luo et al. included independent cohorts from different centers or institutions, whereas Hu et al. used a temporally separate cohort from the same hospital; several other studies relied on bootstrap or random split-sample validation (2, 3, 10). Consequently, the reported C-indices and AUCs should primarily be interpreted as measures of statistical predictive performance rather than evidence of established clinical utility.

In the studies included in this review, graphical calibration assessments were commonly reported, but quantitative calibration measures were limited or inconsistently reported. Independent validation was also uncommon, and assessment of clinical utility was inconsistent. Therefore, the reported discrimination should primarily be interpreted as statistical predictive performance rather than evidence of demonstrated clinical benefit. Before implementation in contemporary LACC practice, existing nomograms require external validation in independent populations receiving current treatment, assessment of calibration across clinically relevant time points and risk ranges, and, where the model is intended to guide treatment or surveillance, evaluation of net clinical benefit.

### 4.6 Limitations of the study

This review has several limitations. First, the included models were developed in heterogeneous cohorts with differences in stage distributions, FIGO staging editions, treatment settings, imaging metrics, radiotherapy techniques, and systemic therapy. Definitions of LACC were not uniform, and two studies included mixed populations containing patients treated with surgery followed by adjuvant radiotherapy, limiting direct comparison across models. Second, validation in genuinely independent cohorts was limited, and the source of validation data varied from independent institutions to temporally separate cohorts from the same hospital and internal split samples. Third, key model-development characteristics—including outcome-event counts, missing-data handling, the effective number of candidate predictor parameters, functional-form specification, and optimism correction—were incompletely reported in several studies. This resulted in more conservative PROBAST judgments, particularly in the Analysis domain, and limited the certainty of cross-study quality comparisons. Fourth, although graphical calibration was frequently reported, quantitative calibration measures such as calibration intercepts, calibration slopes, observed-to-expected ratios, and calibration errors were generally unavailable, and decision-curve analysis was inconsistently performed. Fifth, the review was not prospectively registered. Finally, the rapidly evolving treatment landscape (e.g., INTERLACE, KEYNOTE-A18, IGABT) raises concerns that historical models may misestimate risk in contemporary practice, emphasizing the importance of model updating through recalibration or revision.

## 5 Conclusions

This systematic review shows that published nomograms for LACC patients receiving radiotherapy generally demonstrate moderate-to-good discrimination. Lymph node status, FIGO stage, tumor size, histology, and SCC-Ag were the most consistently incorporated predictors across models. However, substantial heterogeneity in patient populations, treatment settings, staging systems, predictor definitions, and model development methods limits direct comparison across nomograms. Moreover, the limited use of independent external validation and clinical utility assessment restricts their current applicability to routine clinical decision-making. Future studies should prioritize external validation in contemporary treatment cohorts, rigorous assessment of calibration and net clinical benefit, and model updating to account for evolving treatment paradigms. Until such evidence is available, these nomograms should primarily be considered prognostic research tools rather than established instruments for directing individualized treatment or surveillance.

## Data Availability

The original contributions presented in the study are included in the article/**Supplementary Material**. Further inquiries can be directed to the corresponding author.

## References

[1] Wu J Jin Q Zhang Y Ji Y Li J Liu X . Global burden of cervical cancer: current estimates, temporal trend and future projections based on the GLOBOCAN 2022. J Natl Cancer Cent. (2025) 5:322–9. doi: 10.1016/j.jncc.2024.11.006 40693230 PMC12276544

[2] Hu J Shi Q Gong X You T Dai C Chen F . Establishment of a prognostic nomogram based on the clinical and inflammatory parameters as well as acute radiation enteritis for patients with cervical cancer receiving radiotherapy. Front Oncol. (2024) 14:1453837. doi: 10.3389/fonc.2024.1453837 39678508 PMC11638115

[3] Luo WX Ding XM Cheng JM Liu X Zhou HY . Nomogram based on MRI and clinical features to predict progression-free survival in patients with stage IIIC1r cervical squamous cell carcinoma: A two-center study. Clin Radiol. (2024) 79:e1031–9. doi: 10.1016/j.crad.2024.04.010 38749826

[4] Meng Q Wang W Liu X Wang D Zhang F . Nomograms predicting survival of cervical cancer patients treated with concurrent chemoradiotherapy based on the 2018 FIGO staging system. Front Oncol. (2022) 12:870670. doi: 10.3389/fonc.2022.870670 35646645 PMC9130963

[5] Ma H Ma L Yang L Cai M Wang Y Li Y . Construction and evaluation of a prognostic model for cervical cancer based on dynamic hematological and clinical features. BMC Cancer. (2025) 25:207. doi: 10.1186/s12885-025-13595-1 39910481 PMC11800499

[6] Zhang K Sun K Zhang C Ren K Li C Shen L . Using deep learning to predict survival outcome in non-surgical cervical cancer patients based on pathological images. J Cancer Res Clin Oncol. (2023) 149:6075–83. doi: 10.1007/s00432-022-04446-8 36653539 PMC10356676

[7] Zang L Chen Q Zhang X Zhong X Chen J Fang Y . Nomogram predicting overall survival in patients with FIGO II to III squamous cell cervical carcinoma under radical radiotherapy: A retrospective analysis based on 2018 FIGO staging. Cancer Manag Res. (2021) 13:9391–400. doi: 10.2147/cmar.s336892 35002316 PMC8722567

[8] Zang L Chen Q Lin A Chen J Zhang X Fang Y . A prognostic model using FIGO 2018 staging and MRI-derived tumor volume to predict long-term outcomes in patients with uterine cervical squamous cell carcinoma who received definitive radiotherapy. World J Surg Oncol. (2023) 21:210. doi: 10.1186/s12957-023-03116-4 37475053 PMC10360277

[9] Yang X An J Zhang Y Yang Y Chen S Huang M . Prognostic nomograms predicting survival in patients with locally advanced cervical squamous cell carcinoma: The first nomogram compared with revised FIGO 2018 staging system. Front Oncol. (2020) 10:591700. doi: 10.3389/fonc.2020.591700 33194752 PMC7606940

[10] Lin Y Gao Y Weng T . Construction and validation of a MRI‑based radiomic nomogram to predict overall survival in patients with local advanced cervical cancer: A multicenter study. Acad Radiol. (2024) 31:4372–82. doi: 10.1016/j.acra.2024.05.003 38772800

[11] Moons KGM Wolff RF Riley RD Whiting PF Westwood M Collins GS . PROBAST: A tool to assess risk of bias and applicability of prediction model studies: Explanation and elaboration. Ann Intern Med. (2019) 170:W1–W33. doi: 10.7326/m18-1377 30596876

[12] McCormack M Eminowicz G Gallardo D Diez P Farrelly L Kent C . Induction chemotherapy followed by standard chemoradiotherapy versus standard chemoradiotherapy alone in patients with locally advanced cervical cancer (GCIG INTERLACE): an international, multicentre, randomised phase 3 trial. Lancet. (2024) 404:1525–35. doi: 10.1016/s0140-6736(24)01438-7 39419054

[13] Lorusso D Xiang Y Hasegawa K Scambia G Leiva M Ramos-Elias P . Pembrolizumab or placebo with chemoradiotherapy followed by pembrolizumab or placebo for newly diagnosed, high-risk, locally advanced cervical cancer (ENGOT-cx11/GOG-3047/KEYNOTE-A18): overall survival results from a randomised, double-blind, placebo-controlled, phase 3 trial. Lancet. (2024) 404:1321–32. doi: 10.1016/s0140-6736(24)01808-7 39288779

[14] Pötter R Tanderup K Schmid MP Jürgenliemk-Schulz I Haie-Meder C Fokdal LU . MRI-guided adaptive brachytherapy in locally advanced cervical cancer (EMBRACE-I): a multicentre prospective cohort study. Lancet Oncol. (2021) 22:538–47. doi: 10.1016/S1470-2045(20)30753-1 33794207

[15] Riley RD Snell KI Ensor J Burke DL Harrell FE Jr Moons KG . Minimum sample size for developing a multivariable prediction model: PART II‐binary and time‐to‐event outcomes. Stat Med. (2019) 38:1276–96. doi: 10.1002/sim.7992 PMC6519266 30357870

[16] Riley RD Ensor J Snell KI Harrell FE Martin GP Reitsma JB . Calculating the sample size required for developing a clinical prediction model. BMJ. (2020) 368:m441. doi: 10.1136/bmj.m441 32188600

[17] La Salvia A Marcozzi B Manai C Mazzilli R Landi L Pallocca M . Rachel score: a nomogram model for predicting the prognosis of lung neuroendocrine tumors. J Endocrinol Invest. (2024) 47:2575–86. doi: 10.1007/s40618-024-02346-x 38520655

