# Supplementary material for: Predicting outcomes in locally advanced cervical cancer treated with radiotherapy: a systematic review of nomograms
Source: Front Oncol. 2026 Oct 22;16:1940047. doi: 10.3389/fonc.2026.1940047 (PMC13735924; doi:10.3389/fonc.2026.1940047)
Supplement: Supplementary file 1 [file Table1.docx]

**Supplementary Material**

**Supplementary Table S1. Item-level PROBAST signaling-question assessment**

Ratings: Y, yes; PY, probably yes; PN, probably no; N, no; NI, no information.

| **Signaling question** | **Lin 2024** | **Hu 2024** | **Ma 2025** | **Meng 2022** | **Zhang 2023** | **Zang 2021** | **Zang 2023** | **Yang 2020** | **Luo 2024** |
| --- | --- | --- | --- | --- | --- | --- | --- | --- | --- |
| **1.1 Appropriate data source** | Y | Y | Y | Y | Y | Y | Y | Y | Y |
| **1.2 Appropriate inclusions/exclusions** | PY | PY | PY | Y | PY | PY | PN | PY | PY |
| **2.1 Predictors defined/assessed similarly** | Y | Y | Y | Y | Y | Y | Y | Y | Y |
| **2.2 Predictor assessment without outcome knowledge** | PY | PY | PY | PY | PY | PY | PY | PY | PY |
| **2.3 Predictors available at intended prediction time** | Y | PN | PN | Y | Y | Y | Y | Y | Y |
| **3.1 Outcome determined appropriately** | Y | Y | Y | Y | Y | Y | Y | Y | Y |
| **3.2 Prespecified/standard outcome definition** | Y | Y | Y | Y | Y | Y | Y | Y | Y |
| **3.3 Predictors excluded from outcome definition** | Y | Y | Y | Y | Y | Y | Y | Y | Y |
| **3.4 Outcome defined/determined similarly** | Y | Y | Y | Y | Y | Y | Y | Y | Y |
| **3.5 Outcome determined without predictor knowledge** | NI | NI | NI | NI | NI | NI | NI | NI | NI |
| **3.6 Appropriate predictor–outcome interval** | Y | Y | Y | Y | Y | Y | Y | Y | Y |
| **4.1 Reasonable number with outcome** | NI | PY | PN | PY | N | NI | PY | PY | PN |
| **4.2 Continuous/categorical predictors handled appropriately** | PY | PN | PY | PN | PY | PN | PN | PN | PN |
| **4.3 All enrolled participants included** | PY | PY | PY | Y | PY | PY | PN | PY | PY |
| **4.4 Missing data handled appropriately** | NI | PN | PY | NI | NI | NI | N | NI | NI |
| **4.5 Univariable predictor selection avoided** | PY | N | N | N | PN | N | N | N | N |
| **4.6 Data complexities/censoring handled appropriately** | Y | Y | Y | Y | Y | Y | Y | Y | Y |
| **4.7 Relevant model performance evaluated** | Y | Y | Y | Y | Y | Y | Y | Y | Y |
| **4.8 Overfitting/optimism accounted for** | Y | PN | Y | PN | PY | Y | PY | Y | Y |
| **4.9 Final weights correspond to multivariable results** | PY | Y | Y | Y | PY | Y | Y | PN | Y |

PROBAST, Prediction model Risk Of Bias ASsessment Tool

**Supplementary Table S2. Summary of PROBAST risk of bias and applicability assessment**

| **Study** | **Participants** | **Predictors** | **Outcome** | **Analysis** | **Overall RoB** | **Overall applicability concern** |
| --- | --- | --- | --- | --- | --- | --- |
| **Lin 2024** | Low | Low | Unclear | Unclear | Unclear | High |
| **Hu 2024** | Low | High | Unclear | High | High | Unclear |
| **Ma 2025** | Low | High | Unclear | High | High | Unclear |
| **Meng 2022** | Low | Low | Unclear | High | High | Low |
| **Zhang 2023** | Low | Low | Unclear | High | High | High |
| **Zang 2021** | Low | Low | Unclear | High | High | Low |
| **Zang 2023** | High | Low | Unclear | High | High | Low |
| **Yang 2020** | Low | Low | Unclear | High | High | Low |
| **Luo 2024** | Low | Low | Unclear | High | High | Low |

PROBAST, Prediction model Risk Of Bias ASsessment Tool

**Supplementary Table S3. Detailed methodological characteristics, validation, calibration, and clinical-utility assessment**

| **Study** | **Outcome** | **Total N** | **Development N** | **Validation N** | **Outcome events** | **Median follow-up** | **Missing-data handling** | **Candidate predictors/parameters** | **Final predictor parameters** | **Validation method** | **Independent/external cohort source** | **Calibration plot** | **Intercept** | **Slope** | **O:E** | **Calibration error** | **DCA** |
| --- | --- | --- | --- | --- | --- | --- | --- | --- | --- | --- | --- | --- | --- | --- | --- | --- | --- |
| Lin 2024 | OS | 582 | 228 | 98 internal; 256 external | NR | NR | Complete clinical data required; lost to follow-up excluded | NR (radiomic features underwent feature selection) | 3 (age, parametrial invasion, radiomic score) | Random split internal validation + independent multicenter external validation | Hospitals 2–3, n=256 | Yes | NR | NR | NR | NR | Yes |
| Hu 2024 | OS | 288 development + 74 validation | 288 | 74 | 72 deaths in development cohort | 59 mo | 2 lost to follow-up; imputation not reported | NR | 8 | Temporal validation described as external validation | Same hospital, later-period cohort (2020–2021), n=74 | Yes | NR | NR | NR | NR | Yes |
| Ma 2025 | OS | 159 | 159 | — | 31 deaths | 41.37 mo | One patient without on-treatment blood test excluded; all included patients had required blood data | NR | Nomogram 1: 2; Nomogram 2: 7 | Bootstrap internal validation (1,000 resamples) | — | Yes | NR | NR | NR | NR | Yes |
| Meng 2022 | OS; DFS | 1,192 | 795 | 397 | NR | 49.2 mo in development cohort | NR | 7 prespecified clinical characteristics in univariable screening | OS: 5; DFS: 4 | Random split-sample internal validation (2:1) | — | Yes | NR | NR | NR | NR | Not reported for developed nomograms |
| Zhang 2023 | OS | 238 | 165 | 73 test | NR | NR | NR | 11 clinical characteristics; 2,048 deep/pathomic features | Clinical: 2; pathomic: 2 selected deep features; combined model: 3 reported components | Random train/test split; 10-fold CV used for LASSO feature selection | — | Not clearly reported as a formal calibration assessment | NR | NR | NR | NR | NR |
| Zang 2021 | OS | 469 | 469 | — | NR | NR | NR | NR | 4 | Bootstrap resampling (1,000 iterations) | — | Yes | NR | NR | NR | NR | Yes |
| Zang 2023 | OS | 396 | 277 | 119 | NR | 89.77 mo | Patients with missing data and 12 lost to follow-up excluded | NR | 5 | Random split-sample validation (7:3); calibration bootstrap 1,000 resamples | — | Yes | NR | NR | NR | NR | Yes |
| Yang 2020^*^ | OS; PFS | 613 in main analysis* | 613 | — | PFS: 113 progression events; OS events NR | 61.5 mo | Patients with incomplete treatment or medical records excluded; imputation not reported | NR | OS: 6; PFS: 6 | Bootstrap internal validation | — | Yes | NR | NR | NR | NR | NR |
| Luo 2024 | PFS | 144 | 100 | 44 independent validation | Training: 38 progression events; validation: 18 | Training 42 mo; validation 36 mo | Lost to follow-up excluded; imputation not reported | NR | 4 | Bootstrap internal validation (1,000) + independent institutional validation | Institution 2, n=44 | Yes | NR | NR | NR | NR | NR |

**Yang et al.: the abstract reported 618 patients, whereas the Results section/main analysis reported 613; n=613 is used here. NR, not reported; O:E, observed-to-expected ratio; DCA, decision-curve analysis.*

*For Yang et al., the PFS multivariable result for tumor size 4.1–5.9 cm is reproduced as reported in the original publication (HR 1.401, 95% CI 0.967–0.996); the reported HR falls outside the stated 95% CI.*

**Supplementary Table S4. Reports excluded at full-text review (n = 52)**

| **No.** | **First author, year** | **Title** | **Journal** | **PMID** | **Basis for exclusion** |
| --- | --- | --- | --- | --- | --- |
| 1 | Liu L, 2023 | A novel nomogram and risk stratification for early metastasis in cervical cancer after radical radiotherapy | Cancer Med | 37994611 | Author consensus |
| 5 | Chen W, 2023 | Nomogram for prognosis of elderly patients with cervical cancer who receive combined radiotherapy | Sci Rep | 37587180 | Author consensus |
| 6 | Chen W, 2021 | Development and validation of a nomogram for the estimation of response to platinum-based neoadjuvant chemotherapy in patients with locally advanced cervical cancer | Cancer Manag Res | 33603473 | Author consensus |
| 7 | Zheng X, 2021 | Combining intravoxel incoherent motion diffusion weighted imaging and texture analysis for a nomogram to predict early treatment response to concurrent chemoradiotherapy in cervical cancer patients | J Oncol | 34976060 | Author consensus |
| 8 | He JY, 2024 | Development and validation of a prognostic prediction model for cervical cancer patients treated with radical radiotherapy: a study based on TCGA database | Transl Cancer Res | 38737688 | Author consensus |
| 10 | Liu J, 2022 | Outcomes and prognostic factors in patients with locally advanced cervical cancer treated with concurrent chemoradiotherapy | Radiat Oncol | 35978412 | Author consensus |
| 12 | Yan Q, 2024 | MRI radiomics and nutritional-inflammatory biomarkers: a powerful combination for predicting progression-free survival in cervical cancer patients undergoing concurrent chemoradiotherapy | Cancer Imaging | 39449107 | Author consensus |
| 13 | Liu H, 2024 | Development and validation of a 18F-FDG PET/CT radiomics nomogram for predicting progression free survival in locally advanced cervical cancer: a retrospective multicenter study | BMC Cancer | 38291351 | Author consensus |
| 14 | Zhang Y, 2022 | IVIM-DWI and MRI-based radiomics in cervical cancer: prediction of concurrent chemoradiotherapy sensitivity in combination with clinical prognostic factors | Magn Reson Imaging | 35568271 | Author consensus |
| 15 | Feng Y, 2021 | Nomograms predicting the overall survival and cancer-specific survival of patients with stage IIIC1 cervical cancer | BMC Cancer | 33892663 | Author consensus |
| 17 | Wang W, 2018 | Nomogram for predicting para-aortic lymph node metastases in patients with cervical cancer | Arch Gynecol Obstet | 29943132 | Author consensus |
| 18 | Meng X, 2023 | Conditional survival analysis and real-time prognosis prediction for cervical cancer patients below the age of 65 years | Front Oncol | 36698403 | Author consensus |
| 19 | Xu C, 2023 | CT-based radiomics nomogram for overall survival prediction in patients with cervical cancer treated with concurrent chemoradiotherapy | Front Oncol | 38162501 | Author consensus |
| 21 | Sturdza AE, 2021 | Nomogram predicting overall survival in patients with locally advanced cervical cancer treated with radiochemotherapy including image-guided brachytherapy: a Retro-EMBRACE study | Int J Radiat Oncol Biol Phys | 33932530 | Author consensus |
| 22 | Yi J, 2022 | Development and validation of novel nomograms to predict the overall survival and cancer-specific survival of cervical cancer patients with lymph node metastasis | Front Oncol | 35372011 | Author consensus |
| 23 | Luo Y, 2023 | An energy-efficient test and predictive model for recurrence after radiotherapy in localized intermediate and advanced cervical cancer were created using thymidine kinase 1 in conjunction with inflammatory markers and tumor markers | Int J Gen Med | 38089716 | Author consensus |
| 26 | Fan X, 2023 | Prognostic analysis of patients with stage IIIC1p cervical cancer treated by surgery | World J Surg Oncol | 37344912 | Author consensus |
| 28 | Marchetti C, 2018 | Survival nomograms after curative neoadjuvant chemotherapy and radical surgery for stage IB2-IIIB cervical cancer | Cancer Res Treat | 28724282 | Author consensus |
| 29 | Ding R, 2023 | Prognostic models for predicting overall and cancer-specific survival of patients with initially diagnosed metastatic cervical squamous cell carcinoma: a study based on SEER database | Medicine (Baltimore) | 37443499 | Author consensus |
| 30 | Wang H, 2020 | Combined PLT and NE to predict the prognosis of patients with locally advanced cervical cancer | Sci Rep | 32641736 | Author consensus |
| 31 | Gao T, 2024 | Prognostic analysis of stage IIIC1p cervical cancer patients | Front Oncol | 38725620 | Author consensus |
| 32 | Seo Y, 2011 | Nomogram prediction of overall survival after curative irradiation for uterine cervical cancer | Int J Radiat Oncol Biol Phys | 20472352 | Author consensus |
| 33 | Fa XY, 2023 | Development and validation of a nomogram to predict overall survival for cervical adenocarcinoma: a population-based study | Medicine (Baltimore) | 38013281 | Author consensus |
| 34 | Liu Y, 2022 | Identification and validation of serum tumor-markers based nomogram to predict the prognostic value of patients with cervical adenocarcinoma | Curr Probl Cancer | 36270166 | Author consensus |
| 35 | Liu J, 2024 | Competing risk nomogram and risk classification system for evaluating overall and cancer-specific survival in neuroendocrine carcinoma of the cervix: a population-based retrospective study | J Endocrinol Invest | 38170396 | Author consensus |
| 37 | Wang T, 2024 | Risk factor analysis and prediction of para-aortic lymph node metastases in locally advanced cervical cancer | Cancer Med | 39679762 | Author consensus |
| 42 | Ma GF, 2024 | Prediction of recurrence-related factors for patients with early-stage cervical cancer following radical hysterectomy and adjuvant radiotherapy | BMC Womens Health | 38297248 | Author consensus |
| 43 | Wang W, 2019 | Nomograms predicting survival and patterns of failure in patients with cervical cancer treated with concurrent chemoradiotherapy: a special focus on lymph nodes metastases | PLoS One | 30986236 | Author consensus |
| 45 | Zhang S, 2024 | Radiotherapy can improve overall survival in patients with lymph-node positive, high-grade neuroendocrine cervical cancer: construction of two prognostic nomograms to predict treatment outcome | Front Oncol | 39346736 | Author consensus |
| 48 | Zhang Z, 2022 | Prognostic effect of primary recurrence patterns in squamous cervical carcinoma after radical surgery | Front Oncol | 35480094 | Author consensus |
| 49 | Guo M, 2025 | Predictors of para-aortic lymph node metastasis based on pathological diagnosis via surgical staging in patients with locally advanced cervical cancer: a multicenter study | Cancer Lett | 39961430 | Author consensus |
| 53 | Yoon WS, 2017 | Validation of nomograms for survival and metastases after hysterectomy and adjuvant therapy in uterine cervical cancer with risk factors | Biomed Res Int | 28536694 | Author consensus |
| 56 | Liang J, 2022 | Improve individual treatment by comparing treatment benefits: cancer artificial intelligence survival analysis system for cervical carcinoma | J Transl Med | 35765031 | Author consensus |
| 58 | Li Y, 2020 | A prognostic nomogram integrating novel biomarkers identified by machine learning for cervical squamous cell carcinoma | J Transl Med | 32503630 | Author consensus |
| 59 | Lee YJ, 2017 | A postoperative scoring system for distant recurrence in node-positive cervical cancer patients after radical hysterectomy and pelvic lymph node dissection with para-aortic lymph node sampling or dissection | Gynecol Oncol | 28108027 | Author consensus |
| 60 | Li H, 2021 | Radiomic score as a potential imaging biomarker for predicting survival in patients with cervical cancer | Front Oncol | 34485139 | Author consensus |
| 61 | Wei G, 2022 | MRI radiomics in overall survival prediction of local advanced cervical cancer patients treated by adjuvant chemotherapy following concurrent chemoradiotherapy or concurrent chemoradiotherapy alone | Magn Reson Imaging | 35636572 | Author consensus |
| 62 | Xu Z, 2022 | Machine learning of dose-volume histogram parameters predicting overall survival in patients with cervical cancer treated with definitive radiotherapy | J Oncol | 35747125 | Author consensus |
| 63 | Rose PG, 2015 | Nomograms predicting progression-free survival, overall survival, and pelvic recurrence in locally advanced cervical cancer developed from an analysis of identifiable prognostic factors in patients from NRG Oncology/Gynecologic Oncology Group randomized trials of chemoradiotherapy | J Clin Oncol | 25732170 | Author consensus |
| 65 | Yang XL, 2022 | A real-world, population-based study of the trends for incidence and prognosis in high-grade neuroendocrine tumor of cervix | Curr Probl Cancer | 34838343 | Author consensus |
| 68 | Chao B, 2020 | A novel prognostic marker systemic inflammation response index (SIRI) for operable cervical cancer patients | Front Oncol | 32477958 | Author consensus |
| 72 | Zhou Y, 2022 | Multiparametric magnetic resonance imaging-derived radiomics for the prediction of disease-free survival in early-stage squamous cervical cancer | Eur Radiol | 34642807 | Author consensus |
| 73 | Li Y, 2025 | The combined prognostic value of 18F-FDG PET/CT metabolic parameters of immune organs and hematological immune-related markers in patients with locally advanced cervical cancer | Cancer Med | 39912427 | Author consensus |
| 75 | Kidd EA, 2012 | FDG-PET-based prognostic nomograms for locally advanced cervical cancer | Gynecol Oncol | 22735785 | Author consensus |
| 76 | Su X, 2024 | Effect of different treatment modalities on the prognosis of patients with stage IIIC cervical cancer | Front Oncol | 38863632 | Author consensus |
| 77 | Mu W, 2020 | 18F-FDG PET/CT habitat radiomics predicts outcome of patients with cervical cancer treated with chemoradiotherapy | Radiol Artif Intell | 33937845 | Author consensus |
| 78 | Zhang X, 2022 | MRI-based radiomics value for predicting the survival of patients with locally advanced cervical squamous cell cancer treated with concurrent chemoradiotherapy | Cancer Imaging | 35842679 | Author consensus |
| 79 | Kim J, 2021 | Magnetic resonance imaging-based validation of the 2018 FIGO staging system in patients treated with definitive radiotherapy for locally advanced cervix cancer | Gynecol Oncol | 33358037 | Author consensus |
| 80 | Lee WK, 2020 | Prognosis-predicting model based on [18F]fluorodeoxyglucose PET metabolic parameters in locally advanced cervical cancer patients treated with concurrent chemoradiotherapy: multi-center retrospective study | J Clin Med | 32033340 | Author consensus |
| 82 | Zhang X, 2025 | Prognostic value of Naples Prognostic Score in locally advanced cervical cancer patients undergoing concurrent chemoradiotherapy | Biomol Biomed | 39151098 | Author consensus |
| 84 | Fu J, 2024 | The controlling nutritional status score as a new prognostic predictor in patients with cervical cancer receiving radiotherapy: a propensity score matching analysis | BMC Cancer | 39227776 | Author consensus |
| 85 | Shim SH, 2013 | Risk assessment model for overall survival in patients with locally advanced cervical cancer treated with definitive concurrent chemoradiotherapy | Gynecol Oncol | 23063760 | Author consensus |

*No. = record number in the screening sheet. PMID, PubMed identifier. Full-text eligibility was determined by author consensus.*
